# Supplementary material for: An elusive endosymbiont: Does Wolbachia occur naturally in Aedes aegypti?
Source: Ecol Evol. 2020 Jan 16;10(3):1581–91. doi: 10.1002/ece3.6012 (PMC7029055; doi:10.1002/ece3.6012)
Supplement: Supplementary file 1 [file ECE3-10-1581-s001.docx]

**Appendix 1.** Primers used for detecting *Wolbachia* in the *Aedes aegypti* LC laboratory population with molecular assays.

| **Assay** | **Primer specificity** | **Name** | **Sequence (5’-3’)** | **Reference** |
| --- | --- | --- | --- | --- |
| RT/HRM | *Aedes* | mRpS6_F | AGTTGAACGTATCGTTTCCCGCTAC | Lee et al. (2012) |
|  |  | mRpS6_R | GAAGTGACGCAGCTTGTGGTCGTCC |  |
|  | *Aedes aegypti* | aRpS6_F | ATCAAGAAGCGCCGTGTCG | Lee et al. (2012) |
|  |  | aRpS6_R | CAGGTGCAGGATCTTCATGTATTCG |  |
|  | *w*AlbB | wAlbB_F | CCTTACCTCCTGCACAACAA | Axford et al. (2016) |
|  |  | wAlbB_R | GGATTGTCCAGTGGCCTTA |  |
| LAMP (Jasper) | *w*AlbB | WSP_F3 | TGCCTATCACTCCATACGT | Jasper et al. (2019) |
|  |  | WSP_B3 | CTTTAGTAGCTGATACTGTTTCT |  |
|  |  | WSP_FIP | TGCTTGATAAGCAAAACCAAATCCTGGTGCAGCATATATCAGCAA |  |
|  |  | WSP_BIP | AGCTGGTGTTAGTTATGATGTAACCCACCATAAGAACCAAAATAACGAG |  |
|  |  | WSP_FLP | CTTTAACTGCACTAGCTTCTGAAGG |  |
|  |  | WSP_BLP | CCAGAAATCAAGCTTTATGCTGGTG |  |
| TaqMan* | *Wolbachia* | 16S rDNA_F | CCAGCAGCCGCGGTAAT | Mee et al. (2015) |
|  |  | 16S rDNA_R | CGCCCTTTACGCCCAAT |  |
|  |  | Probe | CGGAGAGGGCTAGCGTTATTCGGAATT |  |
| Conventional PCR | *Wolbachia* | 27F | GAGTTTGATCNTGGCTCAG | Wang et al. (2011) |
|  |  | 519R | GTNTTACNGCGGCKGCTG |  |
|  | *w*AlbB | wAlbB_gatB_F | TAAGAATCGCAAGAATTCAC | Baldo et al. (2006) |
|  |  | wAlbB_gatB_R | TGGYAAYTCRGGYAAAGATGA |  |
| LAMP (Kulkarni) | *Wolbachia* | 16S_F3 | CTGGAACTGAGATACGGTC | Kulkarni et al. (2019) |
|  |  | 16S_B3 | TTACGCCCAATAATTCCGA |  |
|  |  | 16S_FIP | TCTTCACTCATGCGGCATGGCAGTGGGGAATATTGGACAA |  |
|  |  | 16S_BIP | AGGAAGATAATGACGGTACTCACAGATAACGCTAGCCCTCTCC |  |
|  |  | 16S_LF | CTGGATCAGGCTTTCGCCC |  |
|  |  | 16S_LB | AGTCCTGGCTAACTCCGTG |  |

* TaqMan assays used VIC (4, 7, 2’-trichloro-7’-phenyl-6-carboxyfluorescein) as the probe reporter dye and TAMRA (6-carboxytetramethylrhodamine) as the quencher dye.

**Appendix 2.** Molecular detection of *Wolbachia* in the *Aedes aegypti* LC laboratory population.

| **Assay** | **Generation** | **Life stage** | **Individuals tested** | **Pools tested (individuals per pool)** | **Percent infected** |
| --- | --- | --- | --- | --- | --- |
| RT/HRM | 3 | 3^rd^ instar larva | 24 | - | 0 |
|  | 4 | 3^rd^ instar larva | 24 | - | 0 |
| LAMP (Jasper) | 3 | 3^rd^ instar larva | 18 | 3 (6) | 0 |
|  | 3 | Adult | 18 | 3 (6) | 0 |
|  | 4 | 3^rd^ instar larva | 18 | 3 (6) | 0 |
|  | 4 | Adult | 18 | 3 (6) | 0 |
| TaqMan | 3 | 3^rd^ instar larva | 24 | - | 0 |
|  | 4 | 3^rd^ instar larva | 24 | - | 0 |
| Conventional PCR (*Wolbachia*) | 3 | 3^rd^ instar larva | 12 | - | 0 |
|  | 4 | Adult | 12 | - | 0 |
| Conventional PCR (*w*AlbB) | 3 | 3^rd^ instar larva | 12 | - | 0 |
|  | 4 | Adult | 12 | - | 0 |
| LAMP (Kulkarni) | 3 | Adult | 12 | 3 (4) | 0 |
|  | 4 | Adult | 12 | 3 (4) | 0 |

**Appendix 3.** Molecular detection of *Wolbachia* in *Aedes aegypti* collected from Ridzuan Condominia, Petaling Jaya (3°04'46.5"N, 101°36'19.9"E) and Section 7, Shah Alam (3°04'10"N, 101°28'58.5"E) in Kuala Lumpur, Malaysia with conventional PCR (Ridzuan Condominia) or RT/HRM (Section 7).

| **Location** | **Date(s) collected** | **Individuals tested** | **Percent infected** |
| --- | --- | --- | --- |
| Ridzuan Condominia | 2013-2014 | 693 | 0 |
| Section 7 | July 4, 2017 | 22 | 0 |
|  | October 5, 2017 | 5 | 0 |
|  | October 30, 2017 | 27 | 0 |
|  | November 27, 2017 | 5 | 0 |
|  | January 3, 2018 | 5 | 0 |
|  | February 5, 2018 | 26 | 0 |
|  | March 26, 2018 | 45 | 0 |
|  | June 4, 2018 | 8 | 0 |
|  | July 11, 2018 | 44 | 0 |
|  | August 7, 2018 | 118 | 0 |
|  | September 5, 2018 | 77 | 0 |
|  | Total | 1075 | 0 |
